# Exploring the relationship between the time until active rehabilitation and length of stay on an adult liver intensive care unit

**DOI:** 10.1186/2197-425X-3-S1-A559

**Published:** 2015-10-01

**Authors:** K Jerrard, S Betteridge, D Spencer, CC Reilly

**Affiliations:** Physiotherapy, Kings College Hospital, London, United Kingdom

## Introduction

It is well documented that patients admitted to Adult Intensive Care Units develop weakness and functional impairment, therefore benefit from early mobilisation and rehabilitation.

The relationship between time until active rehab and length of stay remains to be fully understood in the Intensive Care setting due to the complexity and diversity of this patient cohort.

## Objectives

To establish the length of time taken for patients admitted to the Adult Liver Intensive Care Unit (LITU) to engage in active rehabilitation and to establish common barriers to mobilisation.

## Methods

A service review of adult patients admitted to the LITU. Patient demographics, complexity, time until active rehabilitation and LOS data was recorded.

Active rehabilitation was defined as: active bed exercises and/or active transfer to a chair.

Data was collected by the physiotherapy team on a daily basis. The data was evaluated using simple descriptive statistics (non-parametric) and Spearman’s correlation analysis.

## Results

During January 2015 a total of 49 adult patients were admitted to LITU (32 male and 17 female).

During this time 4 patients died (age (73 (63-83) years), 37 patients were discharged: 5 did not participate in active rehabilitation and 9 remained on the unit at the time of analysis. The data are expressed a median and range.

For these patients the median (range) age was = 52.9 (19 - 83) years and length of stay was 3 (0 - 26) days.

The number of days before patients ‘actively’ engaged in rehab = 1 (0-18)

5 patients (11%) engagement in rehab was > 5 days = 8.5 (6-18) days.

Common barriers to active rehabilitation included sedation, cardiovascular instability, inotropic requirements and neurological impairment.

No correlation was observed between age and length of stay = r = 0.08, p = 0.66. However time until active rehab was correlated with length of stay = r = 0.66, P < 0.001.

## Conclusions

Early active rehabilitation was associated with length of stay in this patient population. Medical instability was the dominant influencing factor increasing time until active rehabilitation.

